# The genome sequence of the Lesser Swallow Prominent, *Pheosia gnoma* (Fabricius, 1777)

**DOI:** 10.12688/wellcomeopenres.19411.1

**Published:** 2023-04-28

**Authors:** Douglas Boyes, Zoe Goate, University of Oxford and Wytham Woods Genome Acquisition Lab, Wellcome Sanger Institute Tree of Life programme, Wellcome Sanger Institute Scientific Operations: DNA Pipelines collective, Tree of Life Core Informatics collective, Darwin Tree of Life Consortium

**Affiliations:** 1 UK Centre for Ecology & Hydrology, Wallingford, England, UK; 2 Wellcome Sanger Institute, Hinxton, England, UK

**Keywords:** Pheosia gnoma, Lesser Swallow Prominent, genome sequence, chromosomal, Lepidoptera

## Abstract

We present a genome assembly from an individual male *Pheosia gnoma* (the Lesser Swallow Prominent; Arthropoda; Insecta; Lepidoptera; Notodontidae). The genome sequence is 271.3 megabases in span. Most of the assembly is scaffolded into 31 chromosomal pseudomolecules, including the Z sex chromosome. The mitochondrial genome has also been assembled and is 17.0 kilobases in length. Gene annotation of this assembly on Ensembl identified 11,628 protein coding genes.

## Species taxonomy

Eukaryota; Metazoa; Ecdysozoa; Arthropoda; Hexapoda; Insecta; Pterygota; Neoptera; Endopterygota; Lepidoptera; Glossata; Ditrysia; Noctuoidea; Notodontidae; Notodontinae; *Pheosia*; *Pheosia gnoma* (Fabricius, 1777) (NCBI:txid988018).

## Background

The Lesser Swallow Prominent, *Pheosia gnoma* (Fabricius, 1777) is a Palearctic species of moth, similar in appearance to the Swallow Prominent ( *Pheosia tremula*) ( Boyes *et al.*, 2021), but is distinguished by a shorter, white wedge-shaped streak at the tornus of the forewing ( Kimber, 2023). *Pheosia gnoma* is widespread and common across southern counties of the British Isles, presenting with a paler-headed phenotype in contrast to localised brown-headed northern populations. Adults fly in two generations, late April to June, and later again in August. *P. gnoma* has been recorded in a variety of habitats, particularly woodland, heathland, moorland, parks and gardens. The larvae feed on silver and downy birch ( *Betula*), overwintering underground as pupae; adults come to light in small numbers ( Barbour *et al.*, 1998).

As the third largest insect order in the world, Lepidoptera are widely used in the study of speciation. Much research has also focused on co-evolutionary dynamics with their host plants and how populations and distributions are changing in relation to climate change ( Chen *et al.*, 2022). The genome of *P. gnoma* was sequenced as part of the Darwin Tree of Life Project, a collaborative effort to sequence all named eukaryotic species in the Atlantic Archipelago of Britain and Ireland. Here we present a complete chromosome-level genome sequence for *P. gnoma*, based on one male specimen from Wytham Woods, Oxfordshire, UK. The genome assembly of *P. gnoma* will contribute to resolving higher-level phylogenetic relationships and better understanding the reasons underpinning species diversification and morphological evolution.

## Genome sequence report

**Figure 1.**
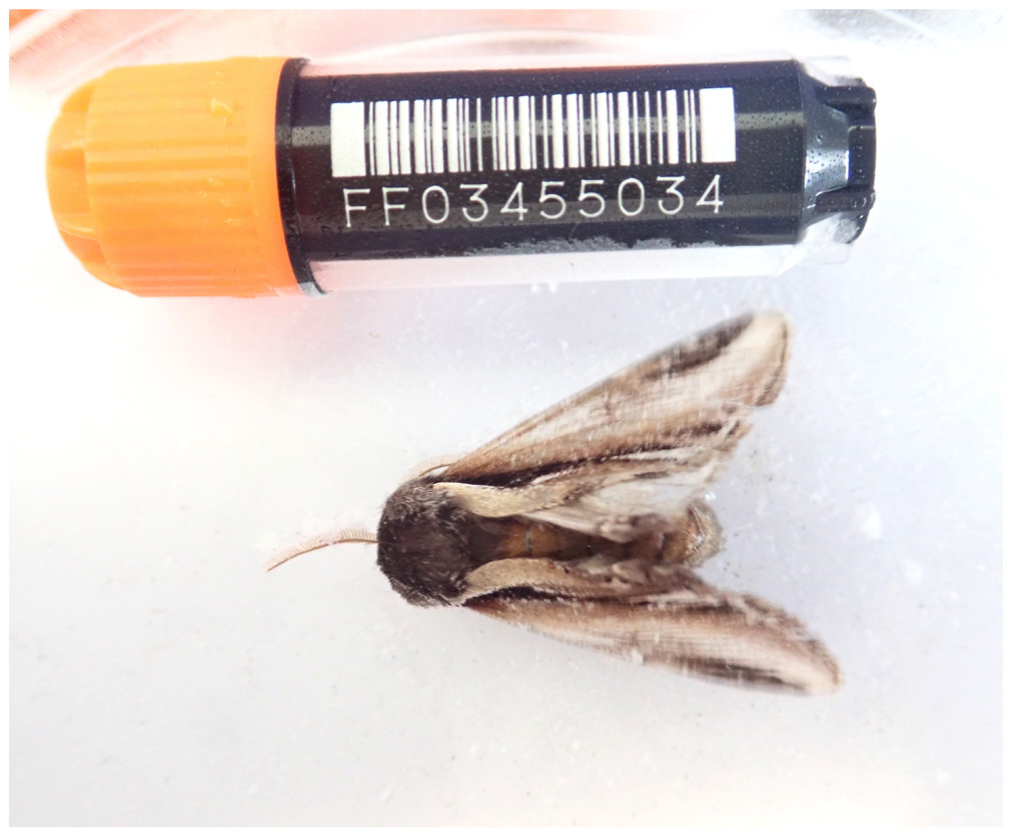
Photograph of the *Pheosia gnoma* (ilPheGnom1) specimen used for genome sequencing.

The genome was sequenced from one male *Pheosia gnoma* ( Figure 1) collected from Wytham Woods, Oxfordshire, UK (latitude 51.77, longitude –1.31). A total of 60-fold coverage in Pacific Biosciences single-molecule HiFi long reads and 148-fold coverage in 10X Genomics read clouds were generated. Primary assembly contigs were scaffolded with chromosome conformation Hi-C data. Manual assembly curation corrected 60 missing joins or mis-joins and removed 6 haplotypic duplications, reducing the scaffold number by 52.5%, and increasing the scaffold N50 by 8.88%.

**Figure 2.**
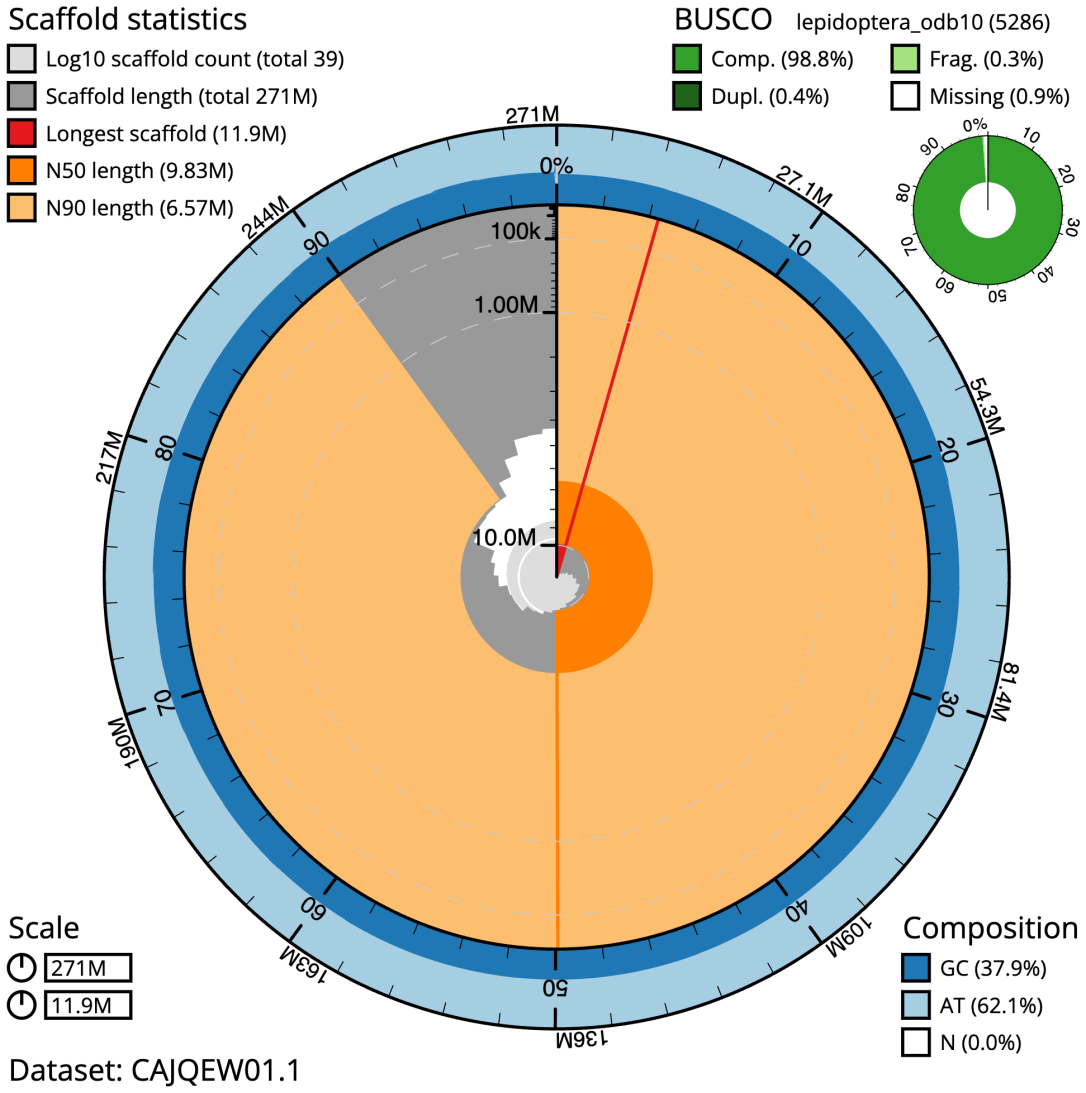
Genome assembly of *Pheosia gnoma*, ilPheGnom1.1: metrics. The BlobToolKit Snailplot shows N50 metrics and BUSCO gene completeness. The main plot is divided into 1,000 size-ordered bins around the circumference with each bin representing 0.1% of the 271,350,489 bp assembly. The distribution of scaffold lengths is shown in dark grey with the plot radius scaled to the longest scaffold present in the assembly (11,949,387 bp, shown in red). Orange and pale-orange arcs show the N50 and N90 scaffold lengths (9,829,711 and 6,572,536 bp), respectively. The pale grey spiral shows the cumulative scaffold count on a log scale with white scale lines showing successive orders of magnitude. The blue and pale-blue area around the outside of the plot shows the distribution of GC, AT and N percentages in the same bins as the inner plot. A summary of complete, fragmented, duplicated and missing BUSCO genes in the lepidoptera_odb10 set is shown in the top right. An interactive version of this figure is available at https://blobtoolkit.genomehubs.org/view/ilPheGnom1.1/dataset/CAJQEW01.1/snail.

The final assembly has a total length of 271.3 Mb in 38 sequence scaffolds with a scaffold N50 of 9.8 Mb ( Table 1). Most (99.93%) of the assembly sequence was assigned to 31 chromosomal-level scaffolds, representing 30 autosomes and the Z sex chromosome. Chromosome-scale scaffolds confirmed by the Hi-C data are named in order of size ( Figure 2– Figure 5; Table 2). While not fully phased, the assembly deposited is of one haplotype. Contigs corresponding to the second haplotype have also been deposited. The mitochondrial genome was also assembled and can be found as a contig within the multifasta file of the genome submission.

**Table 1.** Genome data for *Pheosia gnoma*, ilPheGnom1.1.

Project accession data		
Assembly identifier	ilPheGnom1.1	
Species	*Pheosia gnoma*	
Specimen	ilPheGnom1	
NCBI taxonomy ID	988018	
BioProject	PRJEB44831	
BioSample ID	SAMEA7520513	
Isolate information	ilPheGnom1, male: abdomen (genome sequencing); head and thorax (Hi-C scaffolding and RNA sequencing)	
Assembly metrics *		*Benchmark*
Consensus quality (QV)	55.9	*≥ 50*
*k*-mer completeness	99.99%	*≥ 95%*
BUSCO **	C:98.8%[S:98.5%,D:0.4%], F:0.3%,M:0.9%,n:5,286	*C ≥ 95%*
Percentage of assembly mapped to chromosomes	99.93%	*≥ 95%*
Sex chromosomes	Z chromosome	*localised homologous pairs*
Organelles	Mitochondrial genome assembled	*complete single alleles*
Raw data accessions		
PacificBiosciences SEQUEL II	ERR6412034	
10X Genomics Illumina	ERR6054690– ERR6054693	
Hi-C Illumina	ERR6054689	
PolyA RNA-Seq Illumina	ERR9434972	
Genome assembly		
Assembly accession	GCA_905404115.1	
*Accession of alternate haplotype*	GCA_905404125.1	
Span (Mb)	271.3	
Number of contigs	101	
Contig N50 length (Mb)	7.5	
Number of scaffolds	38	
Scaffold N50 length (Mb)	9.8	
Longest scaffold (Mb)	12.0	
Genome annotation		
Number of protein-coding genes	11,628	
Number of non-coding genes	2,200	
Number of gene transcripts	20,641	

* Assembly metric benchmarks are adapted from column VGP-2020 of “Table 1: Proposed standards and metrics for defining genome assembly quality” from ( Rhie *et al.*, 2021). ** BUSCO scores based on the lepidoptera_odb10 BUSCO set using v5.3.2. C = complete [S = single copy, D = duplicated], F = fragmented, M = missing, n = number of orthologues in comparison. A full set of BUSCO scores is available at https://blobtoolkit.genomehubs.org/view/ilPheGnom1.1/dataset/CAJQEW01.1/busco.

**Figure 3.**
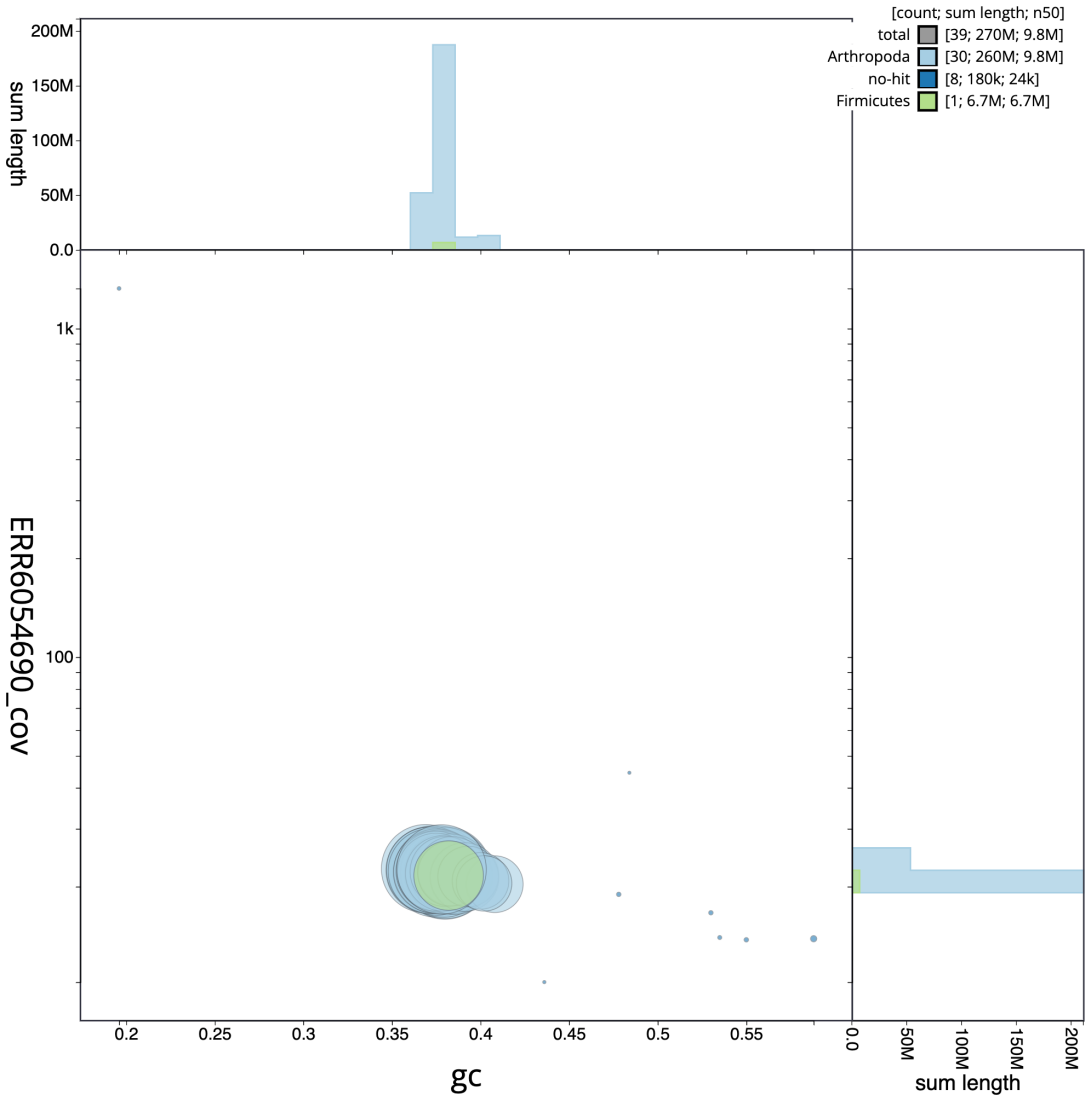
Genome assembly of *Pheosia gnoma*, ilPheGnom1.1: BlobToolKit GC-coverage plot. Scaffolds are coloured by phylum. Circles are sized in proportion to scaffold length. Histograms show the distribution of scaffold length sum along each axis. An interactive version of this figure is available at https://blobtoolkit.genomehubs.org/view/ilPheGnom1.1/dataset/CAJQEW01.1/blob.

**Figure 4.**
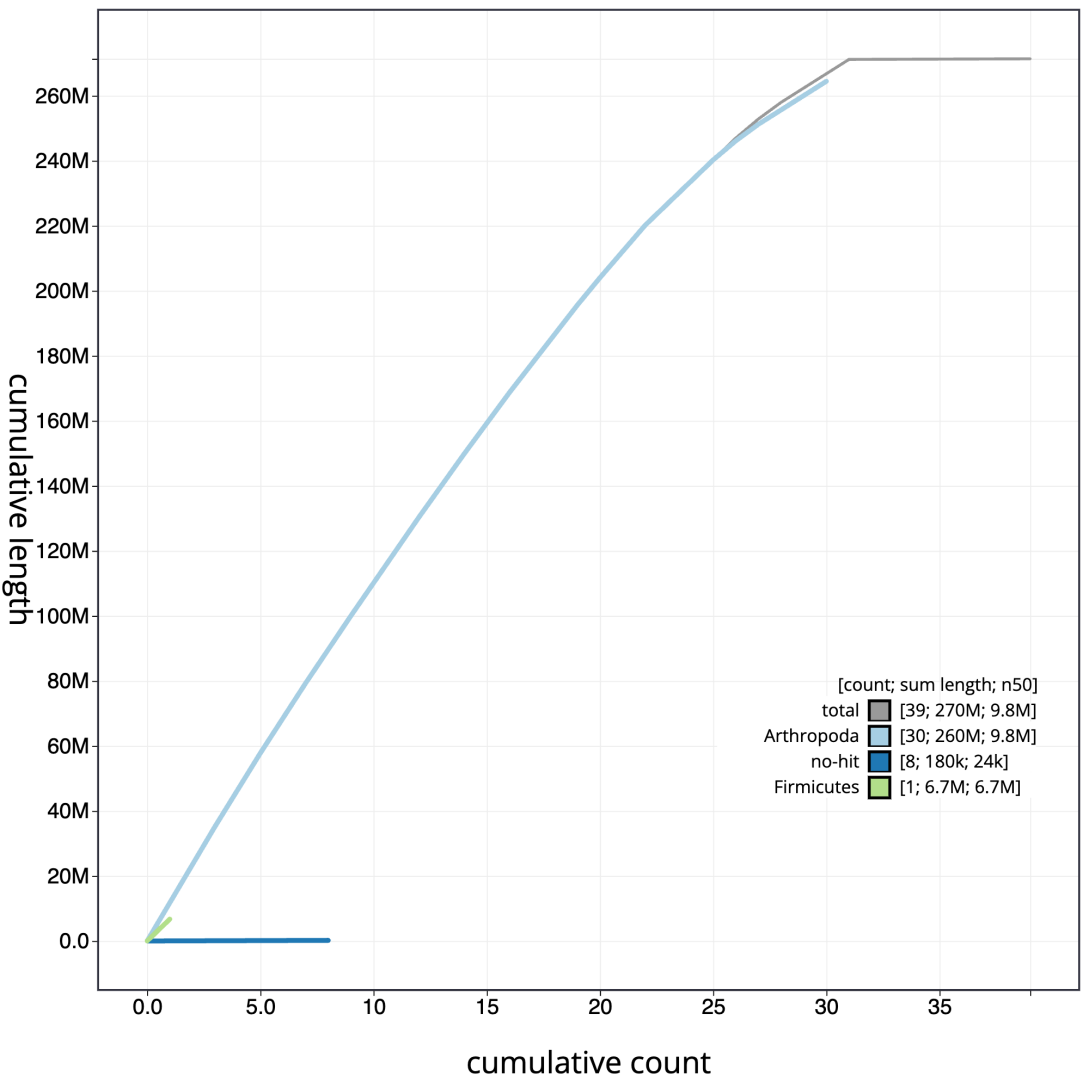
Genome assembly of *Pheosia gnoma*, ilPheGnom1.1: BlobToolKit cumulative sequence plot. The grey line shows cumulative length for all scaffolds. Coloured lines show cumulative lengths of scaffolds assigned to each phylum using the buscogenes taxrule. An interactive version of this figure is available at https://blobtoolkit.genomehubs.org/view/ilPheGnom1.1/dataset/CAJQEW01.1/cumulative.

**Figure 5.**
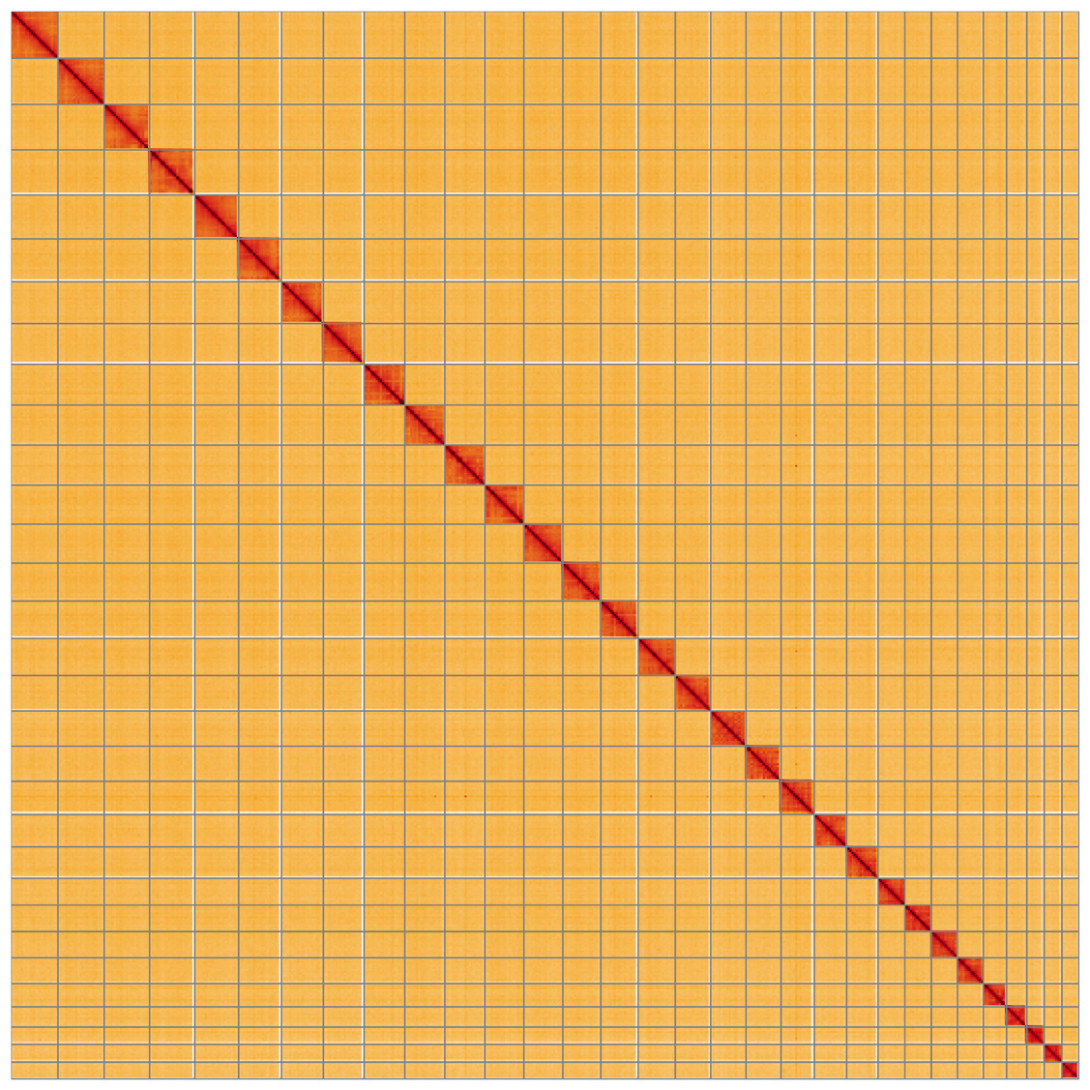
Genome assembly of *Pheosia gnoma*, ilPheGnom1.1: Hi-C contact map of the ilPheGnom1.1 assembly, visualised using HiGlass. Chromosomes are shown in order of size from left to right and top to bottom. An interactive version of this figure may be viewed at https://genome-note-higlass.tol.sanger.ac.uk/l/?d=A0pf4mciQeWJEHevd3RQxA.

**Table 2.** Chromosomal pseudomolecules in the genome assembly of *Pheosia gnoma*, ilPheGnom1.

INSDC accession	Chromosome	Size (Mb)	GC%
FR989894.1	1	11.95	38
FR989895.1	2	11.73	37.5
FR989896.1	3	11.57	38
FR989897.1	4	11.39	37.9
FR989899.1	5	10.87	36.9
FR989900.1	6	10.64	37.2
FR989901.1	7	10.37	37.5
FR989902.1	8	10.35	37.1
FR989903.1	9	10.17	37.1
FR989904.1	10	10.15	37.2
FR989905.1	11	9.96	37.5
FR989906.1	12	9.83	37.6
FR989907.1	13	9.68	37.7
FR989908.1	14	9.47	37.8
FR989909.1	15	9.39	37.4
FR989910.1	16	9.01	38
FR989911.1	17	8.98	37.5
FR989912.1	18	8.9	38.3
FR989913.1	19	8.44	37.5
FR989914.1	20	8.19	38.5
FR989915.1	21	7.96	37.9
FR989916.1	22	6.82	38
FR989917.1	23	6.72	38.2
FR989918.1	24	6.67	38.4
FR989919.1	25	6.57	39.1
FR989920.1	26	5.88	38.3
FR989921.1	27	5.1	39.3
FR989922.1	28	4.48	40.8
FR989923.1	29	4.46	40
FR989924.1	30	4.27	40.2
FR989898.1	Z	11.15	37.8
FR989925.1	MT	0.02	19.6
-	unplaced	0.17	53.4

The estimated Quality Value (QV) of the final assembly is 55.9 with *k*-mer completeness of 99.99%, and the assembly has a BUSCO v5.3.2 completeness of 98.8% (single = 98.5%, duplicated = 0.4%), using the lepidoptera_odb10 reference set ( *n* = 5,286).

Metadata for specimens, spectral estimates, sequencing runs, contaminants and pre-curation assembly statistics can be found at https://links.tol.sanger.ac.uk/species/988018.

## Genome annotation report

The *P. gnoma* genome assembly (GCA_905404115.1) was annotated using the Ensembl rapid annotation pipeline ( Table 1; https://rapid.ensembl.org/Pheosia_gnoma_GCA_905404115.1/Info/Index). The resulting annotation includes 20,641 transcribed mRNAs from 11,628 protein-coding and 2,200 non-coding genes.

**Table 3.** Software tools: versions and sources.

Software tool	Version	Source
BlobToolKit	4.0.7	https://github.com/blobtoolkit/ https://github.com/blobtoolkit/blobtoolkit
BUSCO	5.3.2	https://gitlab.com/ezlab/busco
FreeBayes	1.3.1-17- gaa2ace8	https://github.com/freebayes/ freebayes
gEVAL	N/A	https://geval.org.uk/
Hicanu	2.1	https://github.com/marbl/canu
HiGlass	1.11.6	https://github.com/higlass/higlass
Long Ranger ALIGN	2.2.2	https://support.10xgenomics.com/ genome-exome/software/pipelines/ latest/advanced/other-pipelines
Merqury	MerquryFK	https://github.com/thegenemyers/ MERQURY.FK
MitoHiFi	1	https://github.com/marcelauliano/ MitoHiFi
PretextView	0.2	https://github.com/wtsi-hpag/ PretextView
purge_dups	1.2.3	https://github.com/dfguan/purge_ dups
SALSA	2.2	https://github.com/salsa-rs/salsa

## Methods

### Sample acquisition and nucleic acid extraction

A male *Pheosia gnoma* specimen (individual ilPheGnom1, specimen Ox000389) was collected from collected from Wytham Woods, Oxfordshire (biological vice-county Berkshire), UK (latitude 51.77, longitude –1.31) on 22 May 2020. The specimen was taken from woodland habitat by Douglas Boyes (University of Oxford) using a light trap. The specimen was identified by the collector and snap-frozen on dry ice.

DNA was extracted at the Tree of Life laboratory, Wellcome Sanger Institute (WSI). The ilPheGnom1 sample was weighed and dissected on dry ice with head and thorax tissue set aside for Hi-C and RNA sequencing. Abdomen tissue was cryogenically disrupted to a fine powder using a Covaris cryoPREP Automated Dry Pulveriser, receiving multiple impacts. High molecular weight (HMW) DNA was extracted using the Qiagen MagAttract HMW DNA extraction kit. Low molecular weight DNA was removed from a 20 ng aliquot of extracted DNA using the 0.8X AMpure XP purification kit prior to 10X Chromium sequencing; a minimum of 50 ng DNA was submitted for 10X sequencing. HMW DNA was sheared into an average fragment size of 12–20 kb in a Megaruptor 3 system with speed setting 30. Sheared DNA was purified by solid-phase reversible immobilisation using AMPure PB beads with a 1.8X ratio of beads to sample to remove the shorter fragments and concentrate the DNA sample. The concentration of the sheared and purified DNA was assessed using a Nanodrop spectrophotometer and Qubit Fluorometer and Qubit dsDNA High Sensitivity Assay kit. Fragment size distribution was evaluated by running the sample on the FemtoPulse system.

RNA was extracted from head and thorax tissue of ilPheGnom1 in the Tree of Life Laboratory at the WSI using TRIzol, according to the manufacturer’s instructions. RNA was then eluted in 50 μl RNAse-free water and its concentration assessed using a Nanodrop spectrophotometer and Qubit Fluorometer using the Qubit RNA Broad-Range (BR) Assay kit. Analysis of the integrity of the RNA was done using Agilent RNA 6000 Pico Kit and Eukaryotic Total RNA assay.

### Sequencing

Pacific Biosciences HiFi circular consensus and 10X Genomics read cloud DNA sequencing libraries were constructed according to the manufacturers’ instructions. Poly(A) RNA-Seq libraries were constructed using the NEB Ultra II RNA Library Prep kit. DNA and RNA sequencing were performed by the Scientific Operations core at the WSI on Pacific Biosciences SEQUEL II (HiFi), Illumina HiSeq 4000 (RNA-Seq) and Illumina NovaSeq 6000 (10X) instruments. Hi-C data were also generated from head and thorax tissue of ilPheGnom1 using the Arima2 kit and sequenced on the HiSeq X Ten instrument.

### Genome assembly, curation and evaluation

Assembly was carried out with HiCanu ( Nurk *et al.*, 2020) and haplotypic duplication was identified and removed with purge_dups ( Guan *et al.*, 2020). One round of polishing was performed by aligning 10X Genomics read data to the assembly with Long Ranger ALIGN, calling variants with FreeBayes ( Garrison & Marth, 2012). The assembly was then scaffolded with Hi-C data ( Rao *et al.*, 2014) using SALSA2 ( Ghurye *et al.*, 2019). The assembly was checked for contamination and corrected using the gEVAL system ( Chow *et al.*, 2016) as described previously ( Howe *et al.*, 2021). Manual curation was performed using gEVAL, HiGlass ( Kerpedjiev *et al.*, 2018) and Pretext ( Harry, 2022). The mitochondrial genome was assembled using MitoHiFi ( Uliano-Silva *et al.*, 2022), which runs MitoFinder ( Allio *et al.*, 2020) or MITOS ( Bernt *et al.*, 2013) and uses these annotations to select the final mitochondrial contig and to ensure the general quality of the sequence. To evaluate the assembly, MerquryFK was used to estimate consensus quality (QV) scores and *k*-mer completeness ( Rhie *et al.*, 2020). The genome was analysed within the BlobToolKit environment ( Challis *et al.*, 2020) and BUSCO scores ( Manni *et al.*, 2021; Simão *et al.*, 2015) were calculated. Table 3 contains a list of software tool versions and sources.

### Genome annotation

The Ensembl gene annotation system ( Aken *et al.*, 2016) was used to generate annotation for the *Pheosia gnoma* assembly (GCA_905404115.1). Annotation was created primarily through alignment of transcriptomic data to the genome, with gap filling via protein-to-genome alignments of a select set of proteins from UniProt ( UniProt Consortium, 2019).

### Ethics and compliance issues

The materials that have contributed to this genome note have been supplied by a Darwin Tree of Life Partner. The submission of materials by a Darwin Tree of Life Partner is subject to the Darwin Tree of Life Project Sampling Code of Practice. By agreeing with and signing up to the Sampling Code of Practice, the Darwin Tree of Life Partner agrees they will meet the legal and ethical requirements and standards set out within this document in respect of all samples acquired for, and supplied to, the Darwin Tree of Life Project. All efforts are undertaken to minimise the suffering of animals used for sequencing. Each transfer of samples is further undertaken according to a Research Collaboration Agreement or Material Transfer Agreement entered into by the Darwin Tree of Life Partner, Genome Research Limited (operating as the Wellcome Sanger Institute), and in some circumstances other Darwin Tree of Life collaborators.

## Data Availability

European Nucleotide Archive: *Pheosia gnoma* (lesser swallow prominent). Accession number PRJEB44831; https://identifiers.org/ena.embl/PRJEB44831. ( Wellcome Sanger Institute, 2022) The genome sequence is released openly for reuse. The *Pheosia gnoma* genome sequencing initiative is part of the Darwin Tree of Life (DToL) project. All raw sequence data and the assembly have been deposited in INSDC databases. Raw data and assembly accession identifiers are reported in Table 1.

## Author information

Members of the University of Oxford and Wytham Woods Genome Acquisition Lab are listed here: https://doi.org/10.5281/zenodo.4789928.

Members of the Wellcome Sanger Institute Tree of Life programme are listed here: https://doi.org/10.5281/zenodo.4783585.

Members of Wellcome Sanger Institute Scientific Operations: DNA Pipelines collective are listed here: https://doi.org/10.5281/zenodo.4790455.

Members of the Tree of Life Core Informatics collective are listed here: https://doi.org/10.5281/zenodo.5013541.

Members of the Darwin Tree of Life Consortium are listed here: https://doi.org/10.5281/zenodo.4783558.
