# Supplementary material for: The genome sequence of the Lesser Swallow Prominent, Pheosia gnoma (Fabricius, 1777)
Source: Wellcome Open Res. 2023 Apr 28;8:192. [Version 1] doi: 10.12688/wellcomeopenres.19411.1 (PMC10282565; doi:10.12688/wellcomeopenres.19411.1)
Supplement: Peer review history

# Peer review: Reviewer response for version 1

**DOI:** 10.21956/wellcomeopenres.21504.r58945

**Published:** 2023-06-05

**Authors:** Ryan A St Laurent

**Affiliations:** 1 Smithsonian Institution, Washington, District of Columbia, USA

This manuscript describes the methodology for extraction, library preparation, and sequencing as well as some important statistics for the resulting genome derived from a single male *Pheosia gnoma* (Notodontidae). The manuscript is clearly and economically written. There are a handful of minor comments that I would like to provide as well:

 1. Italicize the Latin name in the Keywords.

 2. Alphabetize the keywords.

 3. As a general rule sentences should not begin with abbreviated genus epithets, but the full genus name spelled out.

 4. In the "Genome sequence report" it is said that "the assembly [is] deposited"  but it is not immediately stated where. I realize this paper is one of many such papers detailing the results of the Darwin Tree of Life initiative, and as such the genomes are all deposited in the same place. However, I think for context it would be helpful to state where a genome or specimen is deposited whenever "deposition" is mentioned, in case a reader only reads this part of the article.

 5. Finally there is an error in the first line of methods, which reads "was collected from collected" which needs to be corrected.

Are sufficient details of methods and materials provided to allow replication by others?

Yes

Is the rationale for creating the dataset(s) clearly described?

Yes

Are the datasets clearly presented in a useable and accessible format?

Yes

Are the protocols appropriate and is the work technically sound?

Yes

Reviewer Expertise:

Evolutionary biology, Lepidoptera systematics, phylogenetics, and taxonomy

I confirm that I have read this submission and believe that I have an appropriate level of expertise to confirm that it is of an acceptable scientific standard.

# Peer review: Reviewer response for version 1

**DOI:** 10.21956/wellcomeopenres.21504.r56678

**Published:** 2023-05-19

**Authors:** Yuttapong Thawornwattana

**Affiliations:** 1 Department of Organismic and Evolutionary Biology, Harvard University, Cambridge, Massachusetts, USA

The authors report a chromosome-level genome assembly of *Pheosia gnoma* (Fabricius, 1777) (Lepidoptera: Notodontidae) obtained from a single male individual using PacBio HiFi long read, 10X Genomics and Hi-C data, produced as part of the Darwin Tree of Life project. This assembly is of high-quality and will be useful to the research community. 

1. However, the rationale for sequencing the genome of this species and the potential uses could be better motivated and more specific (and not just ‘resolving higher-level phylogenetic relationships’ and understanding ‘species diversification’). 
2. The status of the W chromosome was not mentioned. 
3. There was notable a presence of firmicutes bacteria from taxonomic annotation of scaffolds. Would the authors be able to speculate the source of this signal, for example, a contamination or symbiosis (plausible given that the DNA was extracted from the abdomen)?

Are sufficient details of methods and materials provided to allow replication by others?

Yes

Is the rationale for creating the dataset(s) clearly described?

Partly

Are the datasets clearly presented in a useable and accessible format?

Yes

Are the protocols appropriate and is the work technically sound?

Yes

Reviewer Expertise:

Evolutionary genomics, phylogenetics

I confirm that I have read this submission and believe that I have an appropriate level of expertise to confirm that it is of an acceptable scientific standard.
